# Secular Trends in the Epidemiologic Patterns of Thyroid Cancer in China Over Three Decades: An Updated Systematic Analysis of Global Burden of Disease Study 2019 Data

**DOI:** 10.3389/fendo.2021.707233

**Published:** 2021-08-30

**Authors:** Yongze Li, Jianming Piao, Min Li

**Affiliations:** ^1^Department of Endocrinology and Metabolism, Institute of Endocrinology, National Health Commission (NHC) Key Laboratory of Diagnosis and Treatment of Thyroid Diseases, The First Hospital of China Medical University, Shenyang, China; ^2^Department of Neurosurgery, The First Hospital of Jilin University, Changchun, China

**Keywords:** thyroid cancer, epidemiology, China, incidence, mortality

## Abstract

**Background:**

Thyroid cancer is the most common malignant endocrine disease worldwide. The changing epidemiologic pattern of thyroid cancer at the national level in China has remained unknown over the last three decades.

**Methods:**

Following the general analytical strategy used in the Global Burden of Disease Study (GBD) 2019, the age- and sex-specific incidence, mortality, and prevalence rates of thyroid cancer in China were analyzed. Trends in the incidence, mortality, prevalence, and disability-adjusted life years (DALYs) due to thyroid cancer from 1990 to 2019 were assessed by joinpoint regression analysis. Age, period, and cohort effects on incidence were estimated by an age-period-cohort model.

**Results:**

From 1990 to 2019, the age-standardized prevalence and incidence rates significantly increased in both males and females, and the age-standardized mortality rate decreased in females but increased in males. Moreover, the increments in all the age-standardized measures of thyroid cancer in China were higher in males than in females. The age effect showed that those aged 40–44 years had the highest relative risk (RR) among females, and the RR increased with age among males. The incidence increased with time and began to substantially increase in 2009. The cohort effect showed that the incidence decreased in successive birth cohorts.

**Conclusions:**

The burden of thyroid cancer in China showed unexpected patterns that varied by sex, age, and year. Notably, males had higher average annual percentage changes in thyroid cancer incidence and mortality rates than females. More attention should be given to improving the thyroid cancer burden in males in China.

## Introduction

Thyroid cancer is the most pervasive endocrine cancer worldwide (1). In 2017, the number of incident cases and deaths due to thyroid cancer globally were 255,489 and 41,235, respectively (2). Notably, the mortality rate of thyroid cancer decreased during the period of 1990–2017 (3). According to the statistics of GLOBOCAN 2020, thyroid cancer accounted for 3.0% of the 19.3 million new cancer cases and 0.4% of the 10.0 million cancer deaths worldwide in 2020 (4). Compared with the data from GLOBOCAN 2018, both new cases and deaths associated with thyroid cancer increased in 2020 (1). The incidence of thyroid cancer is increasing due to urbanization and economic development. In many middle- and high-income regions, the incidence of thyroid cancer has been growing rapidly, and thyroid cancer has increased sharply in the United States, from 3.6 to 15 cases per 100,000 population from 1973 to 2014 (5). In addition, a widespread and persistent increase in thyroid cancer incidence was also determined in Korea (6).

In China, thyroid cancer is the most common cancer diagnosed before the age of 30 years among women (7). Based on data from the National Central Cancer Registry of China, it was estimated that thyroid cancer accounted for 2.1% (90,000 new cases) of all new cancer cases and 0.2% (6,800 deaths) of all cancer deaths in 2015 (7). A previous study also showed that the incidence rates of thyroid cancer significantly increased annually by 20.1% among females during the period of 2003–2011 (7).

At present, knowledge of thyroid cancer risk factors is still lacking. Age-period-cohort analysis has become a popular method to assess the impact of chronological age, time period, and birth cohort on outcomes, such as disease incidence. The age effect represents the different risks of outcomes associated with different age groups. The period effect reflects the influence of a series of complex historical events and environmental factors. The birth cohort effect reflects the characteristics of each generation and considers risk factors and exposure to environmental factors present in early life (8).

However, updated epidemiologic information about thyroid cancer based on the national population of China is limited. Understanding trends will help guide future research on disease control and prevention strategies. Furthermore, the results of age-period-cohort analyses may provide epidemiologists with important clues or hypotheses about the etiologies of diseases. The Global Burden of Disease (GBD) study is an approach to global descriptive epidemiology (9–11). Therefore, this study aims to analyze data from GBD 2019 to examine the time trend of the burden of thyroid cancer in China and to explore net age, period, and cohort effects under the age-period-cohort framework.

## Materials and Methods

### Study Population and Data Collection

The GBD 2019 study included all available up-to-date sources of epidemiological data and improved standardized methods to provide a comprehensive assessment of health loss considering 369 diseases and injuries and 87 risk factors in 204 countries and territories (12, 13). Details of the methodology used in the GBD 2019 study have been explained in previous studies and are presented in the **Supplement** (12, 13). The GBD 2019 used systematic reviews, survey data, hospital administrative data, disease registries, inpatient and outpatient data, claims, and case notifications as data sources to estimate disease incidence. The original data estimated by GBD for thyroid cancer in China were mainly from the China Disease Surveillance Points (DSP) system, Reporting System of National Maternal and Child Health Surveillance, vital registration data collected by the Chinese Center for Disease Control and Prevention, and the National Central Cancer Registry, which are considered to be nationally representative, as they are based on a national scale (12, 13). The sociodemographic index (SDI) is a comprehensive measurement of education level, income per capita, and fertility rate. As a composite, a location with an SDI of 0 would have a theoretical minimum level of development relevant to health, while a location with an SDI of 1 would have a theoretical maximum level. The SDI index was provided by the Institute for Health Metrics and Evaluation. We compared the trends of the annual SDI and the incidence and mortality rates of thyroid cancer at the global and national levels. Both China-specific and global data were abstracted from the official website (http://ghdx.healthdata.org/gbd-results-tool). Data analysis was completed on March 27, 2021.

The Institutional Review Board of the First Hospital of China Medical University determined that this study did not require approval because it used publicly available data. This study followed the Guidelines for Accurate and Transparent Health Estimates Reporting guidelines for cross-sectional studies (14).

### Statistical Analysis

The age-standardized rates and their average annual percentage changes (AAPCs) were calculated to assess the prevalence, incidence, mortality, disability-adjusted life years (DALYs), years of life lost (YLLs), and years lived with disability (YLDs) of thyroid cancer using linear regression analysis. All the rates are reported per 100,000 population. The 95% uncertainty interval (UI) for each quantity was calculated in our study. Significance in all the analyses was assessed at the 0.05 level, and all hypothesis tests were two-sided.

Joinpoint regression analysis was used to assess trends in the disease burden of thyroid cancer. Joint Command Line Version 4.5.0.1 joinpoint software was provided by the United States National Cancer Institute Surveillance Research Program. This software tracks trends in data over time and then fits the simplest model possible to the data by connecting several different line segments on a logarithmic scale. These segments are known as “joinpoints,” with the simplest model (i.e., 0 joinpoints) being a straight line. As more joinpoints are added, each is tested for significance using a Monte Carlo permutation method. AAPCs were calculated to assess trends, and the Z test was used to assess whether the AAPCs were significantly different from zero. When describing trends, the terms increase or decrease are used when the slope of the trend is statistically significant.

To assess risks in the population in a particular year and the accumulation of health risks since birth, we used the age-period-cohort model. This model allows for the analysis of the independent effects of age, period, and cohort on temporal trends in thyroid cancer incidence. The age-period-cohort model provides a useful parametric framework that complements standard non-parametric descriptive methods. In this model, the collected data were stratified into successive 5-year age groups and consecutive 5-year periods. The incidence rates of thyroid cancer were recorded in successive 5-year age groups (from 5–9 to 75–79 years), consecutive 5-year periods (from 1994 to 2019), and correspondingly consecutive 5-year birth cohorts from 1915–1919 to 2010–2014. The age-period-cohort analysis with the intrinsic estimator method provided estimated coefficients for the age, period, and cohort effects. These coefficients were transformed into exponential values [exp(coef.) = e^coef.^], which denote the incidence relative risk (RR) for a particular age, period, or birth cohort relative to the average level of all ages, periods, or birth cohorts combined. Age-period-cohort analysis was performed using STATA 15.0 software (StataCorp, College Station, TX, USA).

## Results

### Descriptive Analysis

The age-standardized prevalence, incidence, mortality, DALY, YLL, and YLD rates of thyroid cancer by sex in 1990 and 2019 are presented in **Table 1**. Generally, the age-standardized prevalence, incidence, and YLD rates were higher among females than among males in 2019. The age-standardized prevalence, incidence, and mortality rates changed from 6.29 (95% UI, 5.14–7.53) to 16.17 (95% UI, 13.28–19.83), from 1.01 (95% UI, 0.86–1.21) to 2.05 (95% UI, 1.70–2.50), and from 0.42 (95% UI, 0.37–0.53) to 0.39 (95% UI, 0.32–0.45) per 100,000 population in China from 1990 to 2019, respectively (**Table 1**).

**Table 1:** Trends in the thyroid cancer burden between the males and females from 1990 to 2019.

Variable	Age-standardized rate (95% UI)		AAPC1990–2019	Trend 1		Trend 2		Trend 3		Trend 4		Trend 5		Trend 6	
	1990	2019		Year	AAPC	Year	AAPC	Year	AAPC	Year	AAPC	Year	AAPC	Year	AAPC
Males															
Prevalence	2.46 (2.00–3.04)	12.22 (9.29–15.34)	6.70*	1990–1994	2.11*	1994–2002	5.99*	2002–2011	10.03*	2011–2019	2.20*				
Incidence	0.55 (0.45–0.67)	1.74 (1.32–2.16)	4.99*	1990–1994	0.46	1994–2001	3.64*	2001–2007	6.94*	2007–2011	9.67*	2011–2019	1.16*		
Mortality	0.35 (0.29–0.43)	0.52 (0.40–0.64)	2.20*	1990–1994	−0.84*	1994–2001	1.16*	2001–2007	3.28*	2007–2012	5.99*	2012–2019	−2.03*		
DALYs	8.36 (6.89–10.21)	11.64 (8.91–14.29)	1.84*	1990–1994	−1.04*	1994–2000	0.92*	2000–2007	2.40*	2007–2010	6.40*	2010–2013	1.77	2013–2019	−1.61*
YLLs	8.14 (6.67–10.00)	10.82 (8.22–13.45)	1.67*	1990–1994	−1.03*	1994–1999	0.63	1999–2007	2.09*	2007–2010	6.20*	2010–2013	1.63	2013–2019	−1.81*
YLDs	0.21 (0.14–0.30)	0.82 (0.52–1.17)	5.77*	1990–1994	1.17*	1994–2001	4.63*	2001–2006	7.75*	2006–2011	9.87*	2011–2019	1.65*		
Females															
Prevalence	10.42 (8.08–12.91)	20.21 (15.32–26.45)	2.20*	1990–1992	1.28	1992–1999	4.27*	1999–2003	−0.57	2003–2010	3.33*	2010–2016	0.52	2016–2019	3.71*
Incidence	1.50 (1.19–1.86)	2.41 (1.83–3.17)	1.57*	1990–1992	0.37	1992–1999	2.91*	1999–2003	−0.44	2003–2010	2.51*	2010–2016	0.25	2016–2019	3.28*
Mortality	0.50 (0.41–0.63)	0.30 (0.24–0.38)	−1.79*	1990–1998	−1.85*	1998–2004	−1.00*	2004–2015	−2.39*	2015–2019	−0.56*				
DALYs	13.48 (10.98–16.66)	8.11 (6.55–10.07)	−2.00*	1990–2000	−1.18*	2000–2003	−3.28*	2003–2015	−2.32*	2015–2019	−0.05				
YLLs	12.81 (10.33–16.03)	6.92 (5.46–8.71)	−2.38*	1990–1992	−2.27*	1992–1995	−0.71	1995–2000	−1.76*	2000–2007	−3.11*	2007–2015	−2.68*	2015–2019	−0.50*
YLDs	0.67 (0.43–0.97)	1.18 (0.75–1.82)	1.88*	1990–1992	0.79	1992–1999	3.51*	1999–2003	−0.47	2003–2010	2.92*	2010–2016	0.44	2016–2019	3.35*
Total															
Prevalence	6.29 (5.14–7.53)	16.17 (13.28–19.83)	3.52*	1990–1993	2.22*	1993–1999	4.59*	1999–2003	1.27	2003–2011	5.31*	2011–2016	0.93*	2016–2019	3.48*
Incidence	1.01 (0.86–1.21)	2.05 (1.70–2.50)	2.73*	1990–1993	0.89	1993–1998	3.15*	1998–2004	1.74*	2004–2011	4.67*	2011–2016	0.55	2016–2019	2.70*
Mortality	0.42 (0.37–0.53)	0.39 (0.32–0.45)	0.03	1990–1997	−1.13*	1997–2007	0.11	2007–2011	2.36*	2011–2019	−1.32*				
DALYs	10.87 (13.21–9.33)	9.70 (8.11–11.27)	−0.20*	1990–1992	−1.88*	1992–2007	−0.50*	2007–2011	2.09*	2011–2017	−1.32*	2017–2019	−0.19		
YLLs	10.43 (8.97–12.70)	8.70 (7.22–10.22)	−0.44*	1990–1992	−1.91*	1992–2007	−0.71*	2007–2011	1.66*	2011–2019	−1.39*				
YLDs	0.44 (0.30–0.61)	1.00 (0.65–1.45)	3.15*	1990–1993	1.45	1993–1999	3.68*	1999–2003	1.27	2003–2011	4.99*	2011–2016	0.75	2016–2019	3.16*

AAPC, average annual percentage change; DALYs, disability-adjusted life years; YLLs, years of life lost; YLDs, years lived with disability. *indicates P < 0.05 for change in AAPC.

**Figure 1:**
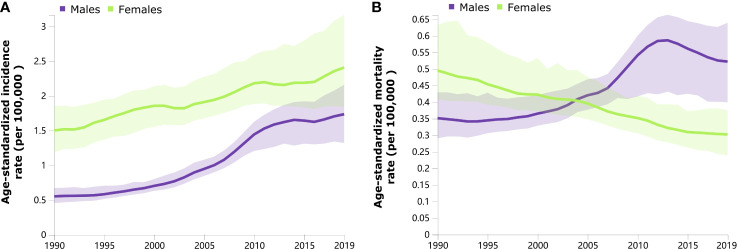
Trends in the age-standardized incidence **(A)** and mortality **(B)** rates of thyroid cancer by sex from 1990 to 2019.

Trends in the sex-specific, age-standardized incidence and mortality rates of thyroid cancer in China from 1990 to 2019 are shown in **Figure 1**. Generally, the age-standardized incidence rates continuously increased from 1990 to 2019 among both males and females (**Figure 1A**). The age-standardized mortality rate in males increased during 1990–2013 and subsequently decreased from 2014 to 2019 (**Figure 1B**). A decreasing trend of the age-standardized mortality rate was observed in females from 1990 to 2019 (**Figure 1B**).

**Figure 2:**
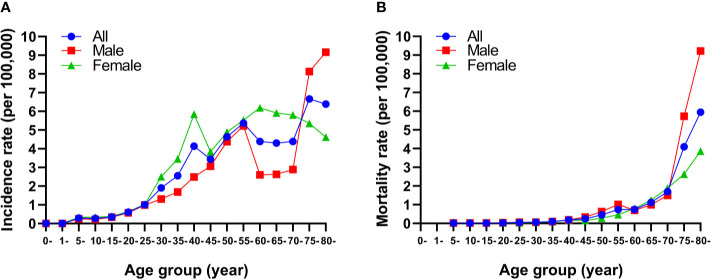
Age-specific incidence **(A)** and mortality **(B)** rates of thyroid cancer by sex in China, 2019.

The highest incidence rate of thyroid cancer among females in 2019 was observed in those aged 60–64 years, followed by those aged 65–69 and 40–44 years (**Figure 2**). For males, the incidence rate of thyroid cancer was higher in those aged 75 years and older, followed by those aged 55–59 years (**Figure 2A**). The mortality rates increased with increasing age in both sexes (**Figure 2B**).

**Figure 3:**
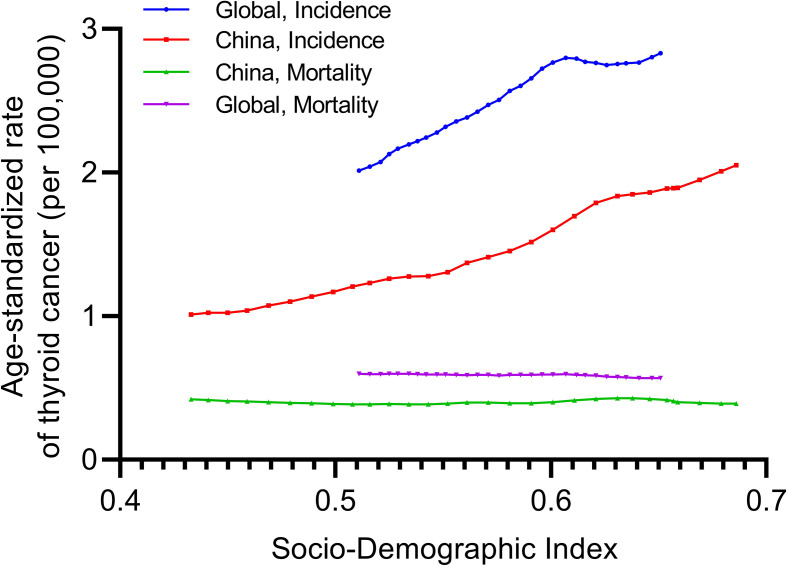
Age-standardized incidence and mortality rates of thyroid cancer globally and in China by sociodemographic index, 1990–2019.

**Figure 3** presents the observed global and China-specific age-standardized incidence and mortality rates from 1990 to 2019 and their associations with the SDI. The expected trend of the age-standardized incidence rate was linear in nature, increasing with increasing SDI values (**Figure 3**). In China, the age-standardized incidence and mortality rates were lower than the global rates based on SDI between 1990 and 2019 (**Figure 3**). Moreover, the trend of the age-standardized mortality rate remained stable, while the SDI values from 1990 to 2019 increased both globally and in China (**Figure 3**).

### Joinpoint Regression Analysis

The AAPCs in the age-standardized rates of thyroid cancer from 1990 to 2019 are presented in **Table 1**. From 1990 to 2019, the age-standardized prevalence, incidence, and YLD rates of thyroid cancer in China increased by 3.52, 2.73, and 3.15%, respectively. Significant AAPC decreases in age-standardized DALY (−0.20%) and YLL (−0.44%) rates were also observed in China (**Table 1**). Moreover, the increments in all the age-standardized measures of thyroid cancer in China were higher in males than in females from 1990 to 2019. The age-standardized mortality, DALYs, and YLLs showed substantial decreases among females (**Table 1**).

### Age-Period-Cohort Analysis With the Intrinsic Estimator Method

**Figure 4:**
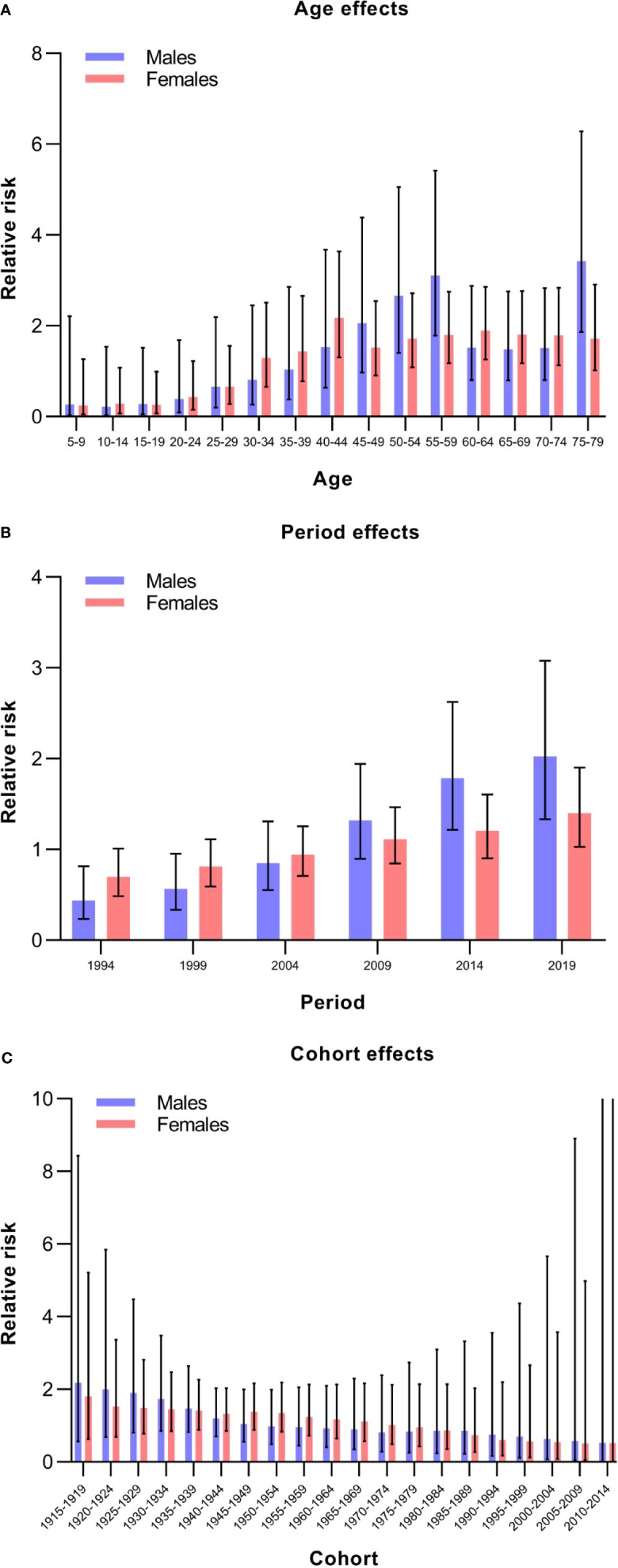
Relative risks of thyroid cancer incidence in China from 1990 to 2019 due to age **(A)**, period **(B)**, and cohort **(C)** effects.

The estimated RRs of thyroid cancer incidence due to age, period, and cohort effects are shown in **Table 2** and **Figure 4**. When the period and cohort effects were controlled for, a higher RR of thyroid cancer incidence was identified in those aged 75–79 years [RR, 3.42; 95% confidence interval (CI), 1.86–6.28], followed by those aged 55–59 years (RR, 3.11; 95% CI, 1.78–5.42) and those aged 50–54 years (RR, 2.66; 95% CI, 1.40–5.06) among males (**Table 2**). For females, the highest age-associated RR, at 2.18 (95% CI, 1.30–3.63), was found in the group aged 40–44 years (**Table 2**). For the period effect, we observed increasing trends of risk of developing thyroid cancer over time among males and females (**Figure 4B**). The RRs associated with period effects showed significant increases from 0.44 (95% CI, 0.23–0.82) in 1994 to 2.02 (95% CI, 1.33–3.08) in 2019 in males and a significantly higher RR, at 1.40 (95% CI, 1.03–1.90), in 2019 for females (**Table 2**). The birth cohort had a non-significant effect on thyroid cancer incidence (**Table 2**). The RRs continuously decreased with later birth cohorts in both males and females (**Figure 4C**).

**Table 2:** Sex-specific relative risks of thyroid cancer incidence in China due to age, period, and cohort effects.

Factor	Males		Females	
	RR (95% CI)	P value	RR (95% CI)	P value
Age				
5–9	0.26 (0.03–2.21)	0.217	0.25 (0.05–1.26)	0.093
10–14	0.21 (0.03–1.54)	0.125	0.28 (0.07–1.08)	0.064
15–19	0.28 (0.05–1.52)	0.139	0.26 (0.07–0.99)	0.048
20–24	0.39 (0.09–1.68)	0.206	0.43 (0.15–1.22)	0.113
25–29	0.66 (0.20–2.19)	0.493	0.66 (0.28–1.56)	0.342
30–34	0.81 (0.27–2.45)	0.706	1.29 (0.66–2.51)	0.459
35–39	1.04 (0.38–2.86)	0.942	1.44 (0.78–2.66)	0.245
40–44	1.53 (0.64–3.67)	0.338	2.18 (1.30–3.63)	0.003
45–49	2.06 (0.97–4.38)	0.061	1.52 (0.90–2.54)	0.114
50–54	2.66 (1.40–5.06)	0.003	1.72 (1.08–2.72)	0.021
55–59	3.11 (1.78–5.42)	<0.001	1.80 (1.17–2.75)	0.007
60–64	1.52 (0.80–2.88)	0.201	1.90 (1.26–2.86)	0.002
65–69	1.48 (0.80–2.76)	0.212	1.80 (1.17–2.76)	0.007
70–74	1.51 (0.80–2.83)	0.2	1.79 (1.13–2.84)	0.013
75–79	3.42 (1.86–6.28)	<0.001	1.72 (1.02–2.91)	0.044
Period				
1994	0.44 (0.23–0.82)	0.009	0.70 (0.48–1.01)	0.056
1999	0.57 (0.34–0.95)	0.032	0.81 (0.59–1.11)	0.192
2004	0.85 (0.55–1.31)	0.457	0.94 (0.71–1.25)	0.681
2009	1.32 (0.90–1.94)	0.16	1.11 (0.85–1.46)	0.447
2014	1.78 (1.21–2.62)	0.003	1.20 (0.90–1.61)	0.206
2019	2.02 (1.33–3.08)	0.001	1.40 (1.03–1.90)	0.032
Cohort				
1915–1919	2.18 (0.56–8.43)	0.26	1.81 (0.63–5.22)	0.275
1920–1924	1.99 (0.68–5.84)	0.208	1.52 (0.69–3.37)	0.299
1925–1929	1.90 (0.81–4.48)	0.141	1.48 (0.78–2.81)	0.226
1930–1934	1.73 (0.86–3.48)	0.126	1.44 (0.84–2.47)	0.181
1935–1939	1.47 (0.82–2.64)	0.198	1.41 (0.88–2.26)	0.154
1940–1944	1.19 (0.70–2.03)	0.511	1.32 (0.86–2.03)	0.208
1945–1949	1.05 (0.55–2.00)	0.885	1.38 (0.88–2.16)	0.167
1950–1954	0.98 (0.49–1.99)	0.958	1.34 (0.83–2.18)	0.231
1955–1959	0.96 (0.45–2.06)	0.911	1.24 (0.72–2.13)	0.435
1960–1964	0.92 (0.40–2.10)	0.837	1.17 (0.64–2.13)	0.606
1965–1969	0.90 (0.35–2.30)	0.819	1.12 (0.57–2.17)	0.747
1970–1974	0.81 (0.28–2.39)	0.707	1.01 (0.49–2.12)	0.969
1975–1979	0.83 (0.25–2.73)	0.764	0.96 (0.43–2.14)	0.917
1980–1984	0.85 (0.24–3.10)	0.809	0.86 (0.35–2.13)	0.753
1985–1989	0.85 (0.22–3.32)	0.818	0.74 (0.27–2.03)	0.558
1990–1994	0.75 (0.16–3.55)	0.721	0.61 (0.17–2.20)	0.447
1995–1999	0.70 (0.11–4.37)	0.702	0.56 (0.12–2.67)	0.468
2000–2004	0.63 (0.07–5.66)	0.683	0.54 (0.08–3.57)	0.525
2005–2009	0.57 (0.04–8.91)	0.689	0.51 (0.05–4.98)	0.559
2010–2014	0.53 (0.01–33.91)	0.762	0.52 (0.01–19.19)	0.724
Deviance	0.43		1.44	
AIC	2.88		3.48	
BIC	−233.56		−232.55	

RR denotes the relative risk of thyroid cancer incidence in a particular age, period, or birth cohort relative to the average level of all age, period, or birth cohort combined. RR, relative risk; CI, confidence interval; AIC, Akaike information criterion; BIC, Bayesian information criterion.

## Discussion

This study found that the age-standardized incidence rate of thyroid cancer significantly increased from 1990 to 2019, with an AAPC of 3.52% in China. The sharp increase in the incidence of thyroid cancer is similar to those in other countries, although this may reflect the increasing use of new imaging techniques for thyroid gland assessment, resulting in the “overdiagnosis” of incidental thyroid cancer (15–20). However, in the absence of disease-stage information, it is impossible to rule out an actual increase in the incidence. In addition, some studies have suggested that “overtreatment” and improvements in examination and diagnosis technologies are only partially responsible for the increase, and additional attention should be given to environmental risk factors (21). Some scholars have noted that possible reasons for the annual increase in the incidence of thyroid cancer include the increase in the number of patients willing to go to a hospital for cancer screening or treatment, the improvements in cancer detection levels, and the increase in the population exposed to radiation. However, the representativeness and population coverage of tumor registries, cancer underreporting, and the quality of registration data will all have an impact on the estimation of thyroid cancer incidence and mortality (21, 22). We observed that the age-standardized mortality rate significantly increased in males but decreased in females. These findings are inconsistent with those of a previous study that reported that thyroid cancer mortality remained stable in females from 2000 to 2011 according to the National Central Cancer Registry of China 2015 (7). These differences in trends in the present and previous studies may be attributed to different sources and degrees of completeness of cancer and mortality data in China. Furthermore, the differences may be related to the duration of the observation period.

In this study, the increase in thyroid cancer burden was significant for every measure, including prevalence, incidence, mortality, DALYs, YLLs, and YLDs, among males. There are several explanations for these differences between men and women. First, women undergo more thyroid examinations because of a higher incidence of benign thyroid disease. In addition, behaviorally, women seek medical evaluations at an earlier stage than men. They also tend to be more involved in medical visits. Furthermore, the differences may be due to the physiological differences between men and women (23–25). Studies have shown that the increase in male mortality may be related to the fact that male patients are mostly diagnosed in the middle and late stages of disease (26). Since 2004, although the incidence rate in females was higher than that in males, the mortality rate in males exceeded that in females and continued to increase, suggesting that improving thyroid cancer prevention in females requires strengthening the early detection and timely treatment of thyroid cancer in males. In addition, the standardized YLD rates in both males and females showed significant increases, indicating that the burden of disease and disability caused by thyroid cancer is increasing.

This study showed that the age-standardized incidence rate of thyroid cancer increased with increasing SDI in China and worldwide. A possible explanation for the increasing incidence might be increased exposure to environmental risk factors, such as ionizing radiation and other carcinogens, and access to diagnostic tools, as well as the increasing SDI. With the increase in the SDI, the stable trend of a lower mortality rate of thyroid cancer may be due to the overall 10-year survival rate for most treatable types of thyroid cancer, at 92–98%, except for incurable types such as anaplastic thyroid carcinoma (27). We also found that thyroid cancer-related mortality increased with increasing age, which may be due to the poor prognosis of thyroid cancer in elderly individuals because of their weakened immune system and underlying diseases (28, 29). Other explanations may be the rapid population aging process and lack of professional medical staff in China.

Generally, the age effect explained why the incidence increased with increasing age among males, while in females, it increased rapidly after the age of 15 and reached a peak in the 40–45 age group. These findings were similar to those of some other studies (30, 31). Thyroid cancer cases in recent decades have mainly occurred in females of menopausal age. A meta-analysis showed that the age of a woman at menopause is an independent risk factor for papillary thyroid cancer (32). A previous study suggested that males with thyroid cancer presented at older ages and had more advanced and aggressive disease courses, which is consistent with our findings that the main age at onset of thyroid cancer in females (40–44 years) was younger than that in males (50–54 years) (33). Considering that these individuals have a large proportion of their expected lifespans remaining and that they contribute substantially to the economy and play a major role in caring for their families, it is necessary to allocate more health resources to high-risk populations and develop tailored programs that can realistically reduce the burden of thyroid cancer.

The period effect is usually influenced by a complex set of historical events and environmental factors. The introduction of highly sensitive ultrasound- and computed tomography-guided fine needle aspiration cytology has contributed to diagnosing many small and previously unknown subclinical thyroid cancers (34, 35). A large number of incidental thyroid nodules have been detected, and most thyroid cancers were diagnosed through pathological examination of these nodules after the introduction of ultrasound for the screening and diagnosis of thyroid cancer (21). A noteworthy increase in the incidence rate of thyroid cancer was observed since 2009 in our study. The American Thyroid Association’s guidelines on the diagnosis and treatment of thyroid cancer have undergone a large number of revisions, and other countries have also developed guidelines during this period, which may be related to the changes noted since 2009. An improved economy is positively correlated with thyroid cancer, and there has been a rapid economic transformation and urbanization in recent decades in China (3). Thus, economic development is one of the probable influential factors for the increasing period-based trends in thyroid cancer incidence (36).

The cohort effect on thyroid cancer incidence revealed continuously decreasing trends in the 1915–1919 to 2010–2014 birth cohorts in both males and females. The possible reason was that the later birth cohorts received a better education and adequate iodine supplementation and had a greater awareness of health and disease prevention than earlier birth cohorts (37). As a country suffering from mild to moderate iodine deficiency, China implemented universal salt iodization (USI) legislation nationally in 1996. A previous study indicated that the correction of iodine deficiency might shift thyroid cancer subtypes toward less malignant forms, which possibly could have contributed to the decreasing cohort trend (38). In addition, Guo et al. found that exposure to the Great Chinese Famine in early life may cause thyroid dysfunction and disorders in adulthood, thereby providing a potential explanation for the decreasing cohort trend (39). However, the reason for the cohort trend in incidence must be interpreted with caution. Improvements are needed for earlier birth cohorts who face a higher risk.

These findings provide epidemiological evidence of the reasons for the increasing thyroid cancer burden. However, this study has some limitations. First, age-period-cohort analysis considers a community as the observed and analyzed unit, which might result in ecological fallacies. Second, although the diagnosis of thyroid cancer with inadequate, insufficiently specific, or unreliable registration was corrected by a redistribution algorithm, the accuracy of diagnosis may still have some unreliability. Third, detailed information about tumor stage or histopathology of thyroid cancer from population-based cancer registries was unavailable. Thus, we could not determine the specific risk factors that affect the prognosis and recurrence of thyroid cancer. In addition, information bias regarding the epidemiologic evaluation of thyroid cancer was inevitable. Therefore, our results in the present study on the epidemiology of thyroid cancer should be interpreted with caution.

## Conclusions

In conclusion, the age-standardized incidence rate of thyroid cancer increased in China among both males and females during the 1990–2019 period. The age-standardized mortality rate significantly increased in males but decreased in females. Notably, males had higher AAPCs in thyroid cancer incidence and mortality rates than females. In addition, females aged 40–44 years had the highest risk of developing thyroid cancer, and males aged 50 years and older had relatively high incidence RRs. The period effect indicated that the risk of developing thyroid cancer continuously increased with time. An improved understanding of risk profiles and onset patterns associated with thyroid cancer could facilitate the early identification of individuals who are at risk of developing thyroid cancer, thereby helping to initiate timely interventions that effectively reduce the thyroid cancer burden. More attention should be given to improving the burden in males in China.

## Data Availability Statement

Publicly available datasets were analyzed in this study. This data can be found here: http://ghdx.healthdata.org/gbd-results-tool.

## Author Contributions

YL and ML conceived and designed the study. YL and JP supervised the study. YL and ML performed the statistical analysis. All authors contributed to the article and approved the submitted version. YL drafted the manuscript. All authors revised the report.

## Funding

This work was supported by the National Natural Science Foundation of China (Grant No. 82000753) and the China Medical University Youth Support Program (Grant No. QGZD2018036).

## Conflict of Interest

The authors declare that the research was conducted in the absence of any commercial or financial relationships that could be construed as a potential conflict of interest.

## Publisher’s Note

All claims expressed in this article are solely those of the authors and do not necessarily represent those of their affiliated organizations, or those of the publisher, the editors and the reviewers. Any product that may be evaluated in this article, or claim that may be made by its manufacturer, is not guaranteed or endorsed by the publisher.
