# Supplementary material for: Secular Trends in the Epidemiologic Patterns of Thyroid Cancer in China Over Three Decades: An Updated Systematic Analysis of Global Burden of Disease Study 2019 Data
Source: Front Endocrinol (Lausanne). 2021 Aug 30;12:707233. doi: 10.3389/fendo.2021.707233 (PMC8435774; doi:10.3389/fendo.2021.707233)
Supplement: Supplementary file 1 [file DataSheet_1.docx]

**Supplement. Overview for Global Burden of Disease 2019**

The Global Burden of Disease (GBD) is an approach to global descriptive epidemiology [1]. It is a systematic, scientific effort to quantify the comparative magnitude of health loss due to diseases, injuries, and risk factors by age, sex, and geographies for specific points in time. IHME serves as the coordinating center for the GBD and affiliated projects. Published in The Lancet in October 2020, GBD 2019 provides for the first time an independent estimation of population, for each of 204 countries and territories and the globe, using a standardized, replicable approach, as well as a comprehensive update on fertility and migration [1]. GBD 2019 incorporates major data additions and improvements, and methodological refinements. Mortality and life expectancy estimates have expanded to a total of 990 locations at the most detailed level, and new causes have been added to the fatal and non-fatal cause lists, for a total of 369 diseases and injuries (http://www.healthdata.org/gbd/about/protocol). GBD 2019 estimated each epidemiological quantity of interest—incidence, prevalence, mortality, years lived with disability (YLDs), years of life lost (YLLs), and disability-adjusted life-years (DALYs)—for 23 age groups; males, females, and both sexes combined; and 204 countries and territories that were grouped into 21 regions and seven super-regions. GBD 2019 location hierarchy now includes all WHO member states. The GBD diseases and injuries analytical framework generated estimates for every year from 1990 to 2019. Diseases and injuries were organised into a levelled cause hierarchy from the three broadest causes of death and disability at Level 1 to the most specific causes at Level 4. Within the three Level 1 causes—communicable, maternal, neonatal, and nutritional diseases; non-com mu-nicable diseases; and injuries—there are 22 Level 2 causes, 174 Level 3 causes, and 301 Level 4 causes (including 131 Level 3 causes that are not further disaggregated at Level 4). 364 total causes are non-fatal and 286 are fatal [1].

**Definition of indicator**

The GBD cause list is organized in a hierarchy. Levels 1 and 2 represent general groupings. The broad group “neoplasms” which includes all malignant and benign neoplasms, is at Level 2 under the Level 1 group “Non-communicable diseases” Level 3 includes 29 cancer groups, and Level 4 includes 37 groups since in Level 4, leukemia, liver cancer, and non-melanoma skin cancer are further subdivided. In this publication, estimates for the GBD cancer groups, for both sexes, for the time from 1980 to 2019, and for the 5-year GBD age groups (0-5; 5-9; etc. until 95+) are presented for 204 countries or territories. Thyroid cancer with ICD10 (C73-C73.9, D09.3, D09.8, D34-D34.9, D44.0) and ICD9 (193-193.9, 226-226.9).

**Data sources**

Data were sought from individual cancer registries or aggregated databases of cancer registry data like Cancer Incidence In Five Continents (CI5). Data were excluded if they were not representative of the coverage population (e.g., hospital-based registries), if they did not cover all malignant neoplasms as defined in ICD9 (140-208) or ICD10 (C00-C96) (e.g., specialty cancer registry), if they did not include data for both sexes and all age groups, if the data were limited to years prior to 1980, or if the source did not provide details on the population covered. Preference was given to registries with national coverage over those with only local coverage, except those from countries where the GBD study provides subnational estimates. Additional metadata for each source are available in the online GBD citation tool, http://ghdx.healthdata.org/gbd-results-tool.

**Modelling**

For most diseases and injuries, processed data are modelled using standardised tools to generate estimates of each quantity of interest by age, sex, location, and year [1]. There are three main standardised tools: Cause of Death Ensemble model (CODEm), spatiotemporal Gaussian process regression (ST-GPR), and DisMod-MR. Previous publications provide more details on these general GBD methods [2-4]. Briefly, CODEm is a highly systematised tool to analyse cause of death data using an ensemble of different modelling methods for rates or cause fractions with varying choices of covariates that perform best with out-of-sample predictive validity testing. DisMod-MR is a Bayesian meta-regression tool that allows evaluation of all available data on incidence, prevalence, remission, and mortality for a disease, enforcing consistency between epidemiological parameters. ST-GPR is a set of regression methods that borrow strength between locations and over time for single metrics of interest, such as risk factor exposure or mortality rates [1].

**Reference**

1. GBD 2019 Diseases and Injuries Collaborators. Global burden of 369 diseases and injuries in 204 countries and territories, 1990-2019: a systematic analysis for the Global Burden of Disease Study 2019. Lancet. 2020 Oct 17;396(10258):1204-1222. doi: 10.1016/S0140-6736(20)30925-9.
2. GBD 2017 Disease and Injury Incidence and Prevalence Collaborators. Global, regional, and national incidence, prevalence, and years lived with disability for 354 diseases and injuries for 195 countries and territories, 1990-2017: a systematic analysis for the Global Burden of Disease Study 2017. Lancet. 2018 Nov 10;392(10159):1789-1858. doi: 10.1016/S0140-6736(18)32279-7.
3. GBD 2017 Causes of Death Collaborators. Global, regional, and national age-sex-specific mortality for 282 causes of death in 195 countries and territories, 1980-2017: a systematic analysis for the Global Burden of Disease Study 2017. Lancet. 2018 Nov 10;392(10159):1736-1788. doi: 10.1016/S0140-6736(18)32203-7.
4. GBD 2017 Diet Collaborators. Health effects of dietary risks in 195 countries, 1990-2017: a systematic analysis for the Global Burden of Disease Study 2017. Lancet. 2019 May 11;393(10184):1958-1972. doi: 10.1016/S0140-6736(19)30041-8.
